# Potent Induction of Envelope-Specific Antibody Responses by Virus-Like Particle Immunogens Based on HIV-1 Envelopes from Patients with Early Broadly Neutralizing Responses

**DOI:** 10.1128/JVI.01343-21

**Published:** 2022-01-12

**Authors:** Carolina Beltran-Pavez, Ilja Bontjer, Nuria Gonzalez, Maria Pernas, Alberto Merino-Mansilla, Alex Olvera, Jose M. Miro, Christian Brander, Jose Alcami, Rogier W. Sanders, Victor Sanchez-Merino, Eloisa Yuste

**Affiliations:** a Institute of Health Carlos III, ISCIII, National Microbiology Centre, Madrid, Spain; b University of Barcelona, Faculty of Pharmacy and Food Science, Barcelona, Spain; c Department of Medical Microbiology, Amsterdam Institute for Infection and Immunity, Amsterdam UMC, University of Amsterdam, Amsterdam, The Netherlands; d Department of Microbiology and Immunology, Weill Medical College of Cornell University, New York, New York, USA; e IrsiCaixa, AIDS Research Institute, Hospital Germans Trias i Pujol, Badalona, Spain; f Universitat de Vic-Universitat Central de Catalunya, Vic, Spain; g Infectious Diseases Service, Hospital Clinic-IDIBAPS. University of Barcelona, Barcelona, Spain; h ICREA, Barcelona, Spain; Emory University

**Keywords:** HIV-1, neutralizing antibodies, vaccines, virus-like particles

## Abstract

Longitudinal studies in HIV-1-infected individuals have indicated that 2 to 3 years of infection are required to develop broadly neutralizing antibodies. However, we have previously identified individuals with broadly neutralizing activity (bNA) in early HIV-1 infection, indicating that a vaccine may be capable of bNA induction after short periods of antigen exposure. Here, we describe 5 HIV-1 envelope sequences from individuals who have developed bNA within the first 100 days of infection (early neutralizers) and selected two of them to design immunogens based on HIV-1-Gag virus-like particles (VLPs). These VLPs were homogeneous and incorporated the corresponding envelopes (7 to 9 μg of gp120 in 10^10^ VLPs). Both envelopes (Envs) bound to well-characterized broadly neutralizing antibodies (bNAbs), including trimer-specific antibodies (PGT145, VRC01, and 35022). For immunogenicity testing, we immunized rabbits with the Env-VLPs or with the corresponding stabilized soluble envelope trimers. A short immunization protocol (105 days) was used to recapitulate the early nAb induction observed after HIV-1 infection in these two individuals. All VLP and trimeric envelope immunogens induced a comparably strong anti-gp120 response despite having immunized rabbits with 30 times less gp120 in the case of the Env-VLPs. In addition, animals immunized with VLP-formulated Envs induced antibodies that cross-recognized the corresponding soluble stabilized trimer and vice versa, even though no neutralizing activity was observed. Nevertheless, our data may provide a new platform of immunogens, based on HIV-1 envelopes from patients with early broadly neutralizing responses, with the potential to generate protective immune responses using vaccination protocols similar to those used in classical preventive vaccines.

**IMPORTANCE** It is generally accepted that an effective HIV-1 vaccine should be able to induce broad-spectrum neutralizing antibodies. Since most of these antibodies require long periods of somatic maturation *in vivo*, several groups are developing immunogens, based on the HIV envelope protein, that require complex and lengthy immunization protocols that would be difficult to implement in the general population. Here, we show that rabbits immunized with new envelopes (VLP formulated) from two individuals who demonstrated broadly neutralizing activity very early after infection induced specific HIV-1 antibodies after a short immunization protocol. This evidence provides the basis for generating protective immune responses with classic vaccination protocols with vaccine prototypes based on HIV envelope sequences from individuals who have developed early broadly neutralizing responses.

## INTRODUCTION

Classical vaccines are based on the induction of neutralizing antibodies, and, in HIV infection, envelope is the protein responsible for induction of these antibodies. However, HIV envelope has evolved to evade immune recognition through different mechanisms such as high variability, coating with carbohydrates, and steric occlusion. As a result, immunization with recombinant gp120 does not induce an effective neutralizing antibody response against different HIV isolates (1). Confirmation that induction of a protective antibody response by vaccination is feasible has been provided by a recent study that showed protection against the simian immunodeficiency virus after passive transfer of purified IgGs from prototype Ad26/Env-vaccinated rhesus macaques (2).

Some studies have found that between 10 and 25% of HIV-1-infected individuals demonstrated broadly neutralizing activity, including against other HIV subtypes (3–6). The antibody response has been thoroughly studied in these individuals, new broadly neutralizing antibodies (bNAbs) have been isolated, and the corresponding epitopes have been identified and mapped to gp160. The identification of these elite neutralizer individuals (EN) represents a central element in the isolation and characterization strategy of new antibodies with a wide and powerful neutralization capacity. This strategy has proven to be especially useful in defining new epitopes preserved in gp160 that can be integrated into the design of potential vaccine immunogens (7–9). In fact, several recent studies have used this knowledge to design trimers that mimic native envelope conformation and are indeed able to induce autologous nAb responses. Subsequent structure-based modifications of these stabilized trimers have resulted in immunogens capable of inducing neutralizing antibodies against tier 2 viruses, which are more difficult to neutralize and have greater clinical relevance than a tier 1 or autologous virus (10, 11). Despite these improvements, the breadth of the induced responses and their duration *in vivo* are limited.

Cryogenic electron microscopy (cryo-EM) structures of native full-length trimers, with no stabilizing mutations, in complexes with broadly neutralizing antibodies have revealed a considerable plasticity that is exploited by several antibodies (12). Considering this flexibility, one of the potential drawbacks of using stabilized trimers is that they fix the structure in a given conformation that can limit access to epitopes relevant to bNAbs induction, such as the V2 bNAb cluster. To solve this limitation, certain disulfide bonds have been incorporated into the design to stabilize the V1 and V2 domains in a conformation that improves the presentation of V2 bNAb epitopes (13). While these can indeed improve bNAbs induction, fixing a specific protein conformation can alter the exposure of other epitopes of interest in remote areas within the protein.

In the case of HIV, prototypes based on virus-like particles (VLP)-formulated Env immunogens are presented to the immune system in a flexible membrane-associated conformation that provides the environment most similar to that of the virion. In addition, VLP-formulated HIV Envs would provide a multivalent presentation of the immunogen that could improve its immunogenicity. This has been observed when a soluble, stabilized HIV Env trimer is formulated on ferritin nanoparticles and in other nanoparticle-based vaccines, such as human papillomavirus (HPV), hepatitis B virus (HBV), Epstein-Barr virus (EBV), influenza, and malaria vaccines (11, 14–18). The good results obtained in nanoparticle-based influenza and malaria vaccines support the hypothesis that multivalent presentation of an immunogen may improve its immunogenicity.

In order to mimic the natural induction of bNAbs in elite neutralizer individuals, several groups have developed immunogens based on the envelope from individuals who have demonstrated the ability to induce broadly neutralizing responses. Since most of these antibodies require long periods of somatic hypermutation and are thus usually generated late in infection, HIV-Env immunogens have been mostly based on envelopes from patients in chronic infection. However, recreating envelope structures that induce broadly neutralizing responses in a later stage of infection would call for complex and lengthy immunization strategies that would be difficult to implement to the general population.

Given that we have identified individuals with broadly neutralizing responses within the first 6 months of their infection, we hypothesized that immunogens based on their envelope sequences in early infection could be used as immunogens to trigger bNAbs induction. To verify this hypothesis, we have generated and characterized immunogens based on VLP, formulated with early infection Envs, and tested these in animal models. Considering that an effective HIV vaccine, in addition to eliciting an efficient humoral response, will possibly need to induce a robust T cell immunity (19), we have incorporated a variant of the HIV Gag protein that has been optimized as a T cell immunogen and assembles particles comparable to HIV virions in our VLP design (20).

## RESULTS

### Characterization of selected individuals with broadly neutralizing responses during early recent infection (<100 days postinfection).

We have previously identified HIV-1-infected individuals with broadly neutralizing activity (bNA) within the first 6 months of infection (21). This study included 88 individuals with early recent infection (<100 days postinfection [dpi]). Based on their neutralization breadths in a screening for cross-neutralizing sera (21), five of these individuals were selected for the present study. Sera from all five neutralized more than 50% of the viruses from the minipanel at a 1:200 dilution, and two of these participants (DET 887 and DET 936) neutralized four subtypes at 96 and 82 dpi, respectively (Table 1). Complete neutralization curves at the same time point could only be performed for DET 887 due to sera availability. For the rest of the samples, they could be completed using subsequent samples from the same individuals still corresponding to early periods of infection (55 dpi for DET 763, 47 dpi for DET 892, 159 dpi for DET 902, and 82 dpi for DET 936). These curves confirmed the presence of broadly neutralizing responses, and the corresponding 50% infective dose (ID_50_) values are shown in Table 2.

**TABLE 1:** Sera neutralization data for selected recently infected patients corresponding to the date they were characterized as broadly neutralizing^*a*^

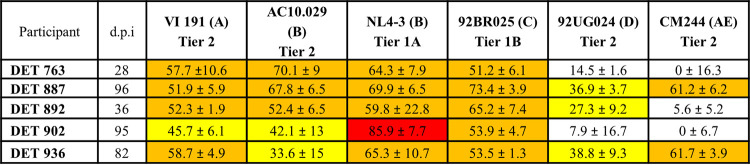

a Percentages of neutralization at a 1:200 serum dilution. White box indicates <50% neutralization, yellow box indicates ≥25% and <50% neutralization, orange box indicates ≥50% neutralization and <75% neutralization, and red box indicates ≥75% neutralization.

**TABLE 2:** Plasma neutralization data for individuals with broadly neutralizing activity in recent infection^*a*^

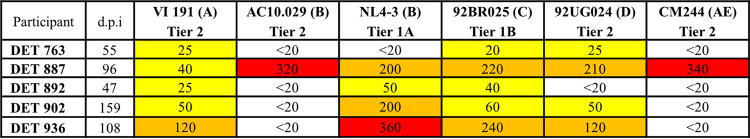

a White box indicates ID_50_ <20, yellow box indicates ID_50_ ≥20 and <100, orange box indicates ID_50_ ≥100 and <200, and red box indicates ID_50_ ≥300.

Demographic and clinical characteristics of these early neutralizers are shown in Table 3. All participants were antiretroviral therapy (ART) untreated, Iberian Peninsula natives, and classified within Fiebig stage V. Four individuals were male, and one was female (DET 892). Heterosexual transmission was reported for DET 892, while men who have sex with men (MSM) accounted for the rest. CD4-positive (CD4^+^) T cell counts, viral loads (VL), and times of infection are also indicated.

**TABLE 3:** Patient clinical characteristics

Parameter	Data for:				
	DET 763	DET 887	DET 892	DET 902	DET 936
Days postinfection^*a*^	29	96	36	95	82
Gender	M	M	F	M	M
Age (yrs)	30	42	39	31	44
Origin	Spain	Spain	Portugal	Spain	Spain
Transmission route	MSM	MSM	Heterosexual	MSM	MSM
Test for recent infection^*b*^	<180 days	<180 days	<180 days	<180 days	<180 days
Western blotting result (p31 band)	Negative	Negative	Negative	Negative	Negative
Symptomatic primary infection	Yes	Yes	No	Yes	No
Fiebig stage	V	V	V	V	V
Subtype (RT/protease)	B	B	B	B	B
Viral tropism^*c*^	R5	Dual	Dual	R5	R5
CD4^+^^*d*^ (cells/μl)	435	653	884	340	714
CD8^+^^*d*^ (cells/μl)	7629	658	1300	1190	448
CD4^+^/CD8^+^^*d*^	0.06	0.99	0.68	0.29	1.59
Plasma VL (no. of copies/ml)^*d*^	500001	76311	10930	32946	20279
Plasma VL (log_10_)^*d*^	5.7	4.88	4.04	4.52	4.31

a The estimated time of infection was determined according to the previously described algorithm (32).

b The test for recent infection used was BED capture enzyme immunoassay (BED-CEIA) (modified version of the Vironostika HIV-1 enzyme immunoassay; bioMérieux Durham, NC, USA) (56).

c Viral tropism has been determined by Trofile assay, genotypic sequencing in HIV RNA, or in proviral HIV-1 DNA in PBMCs.

d At the time of sample collection.

### Epitope mapping.

**FIG 1:**
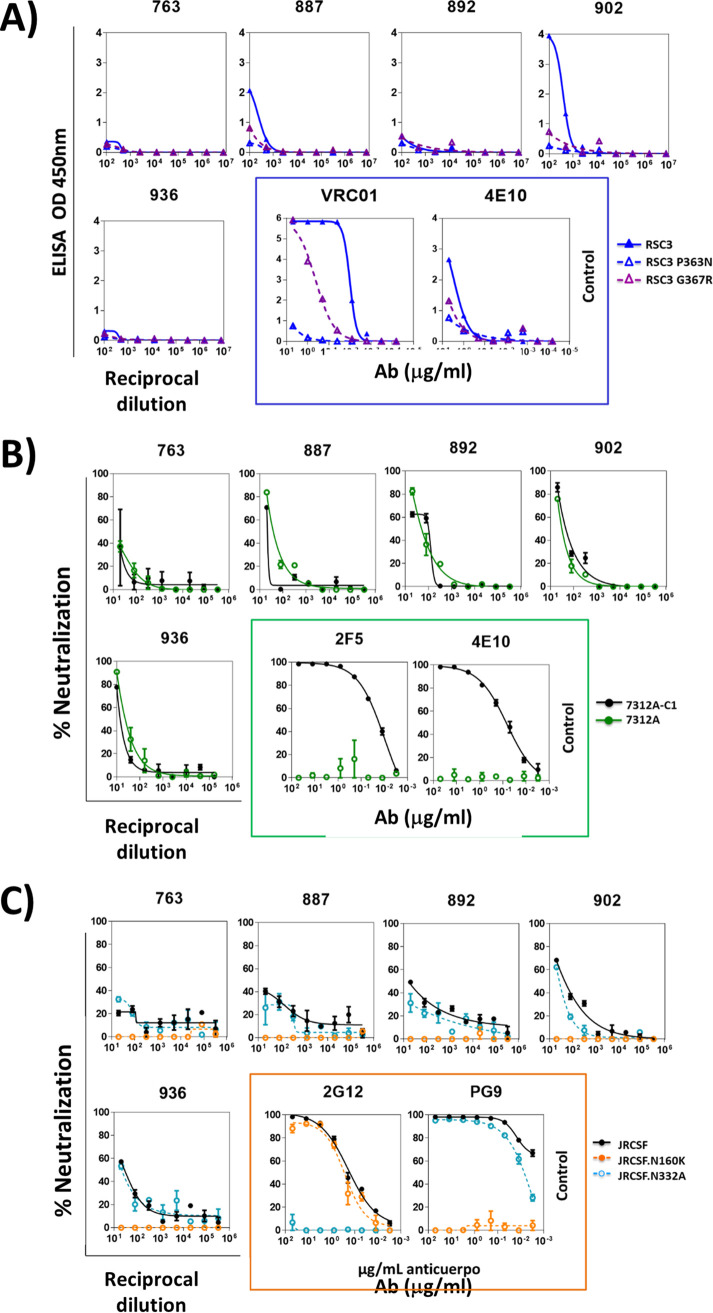
Epitope mapping of the sera from 5 early neutralizers. (A) Sera with antibodies against CD4bs were detected by ELISA with the following probes: resurfaced stabilized cores RSC3 and RSC3 P363N. Monoclonal antibodies 4E10 and VRC01 have been used as controls and are indicated by a blue box. (B) Sera with neutralizing antibodies against the MPER are detected by neutralization assays with viruses 7312A-C1 and 7312A. Monoclonal antibodies 2F5 and 4E10 have been used as controls and are indicated by a green box. (C) Sera with neutralizing antibodies against glycan-dependent motifs in the V2 and V3 loops are detected by neutralization assays with JRCSFwt and the corresponding N160K and N332A mutants (V2 and V3, respectively). Monoclonal antibodies 2G12 and PG9 have been used as controls and are indicated by an orange box.

The epitopes targeted by the neutralizing antibodies present in serum were mapped in samples obtained at 55, 96, 47, 159, and 108 dpi for DET 763, DET 887, DET 892, DET 902, and DET 936, respectively. A decrease in plasma neutralization against JRCSF.N160K compared to the neutralization of wild-type JRCSF suggested the presence of neutralizing antibodies directed to V2 glycans, consistent with previously published data in recent HIV-1 infection (Fig. 1C) (21). Antibodies directed to CD4bs were detected in DET 902 (Fig. 1A). Antibodies directed to V3 glycan-dependent and membrane-proximal external region (MPER)-dependent epitopes were not detected in any of the tested sera (Fig. 1B and C).

### Characterization of HIV-1 envelopes from the selected early neutralizers and selection of envelopes for VLP-based immunogen design.

**FIG 2:**
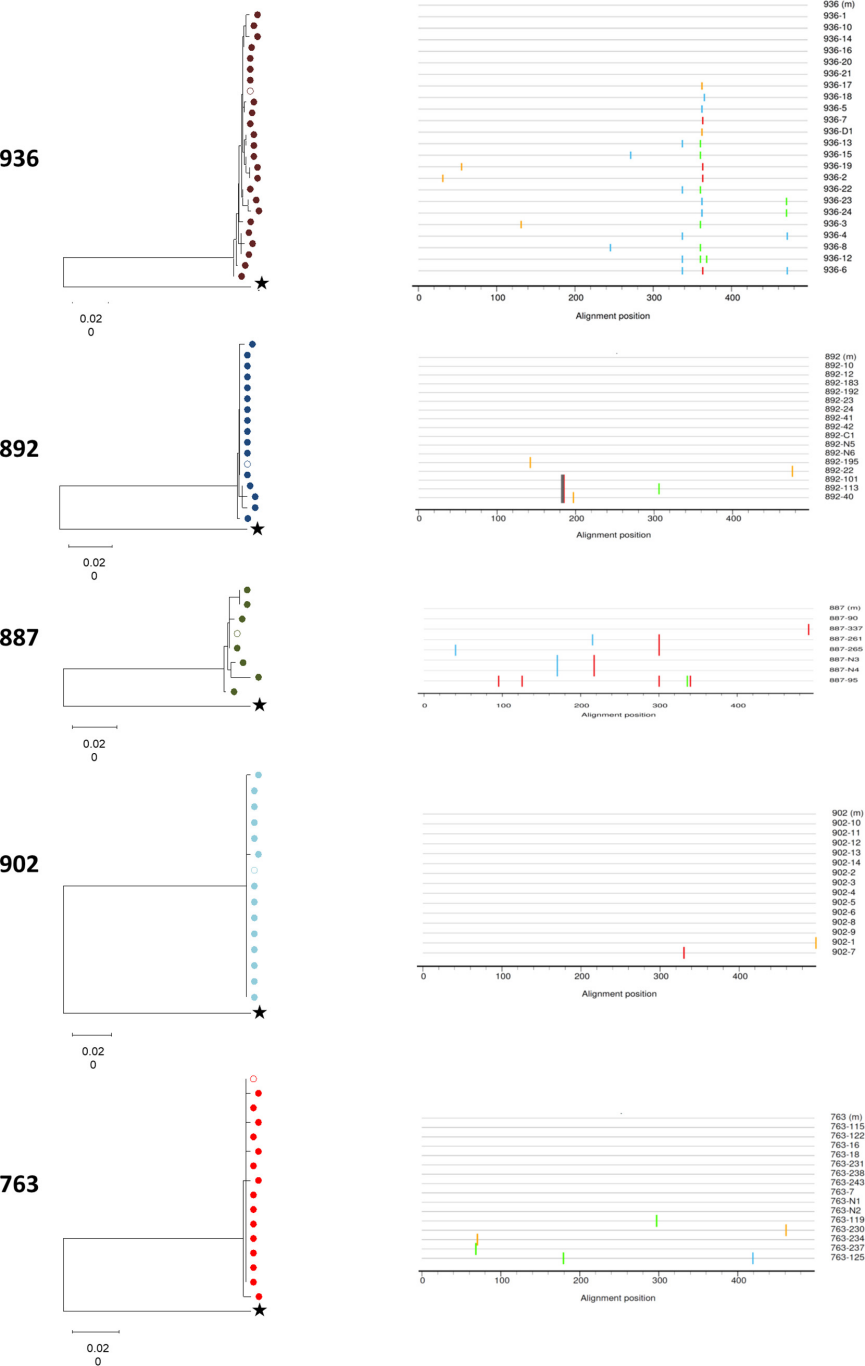
Sequence phylogenetic analysis (C2-V5 region) of the virus circulating in plasma. (Left) Neighbor-joining (NJ) phylogenetic tree of the C2-V5 Env region was constructed with 81 sequences obtained from plasma viral RNA. Each patient is color-coded, and the empty circle represents the corresponding “master sequence.” Outgroup sequence (B.FR.83.HXB2 LAI IIIB BRU K03455) is represented by star. Branch lengths are presented as the number of substitutions per site. All patients’ sequences grouped with bootstrap values higher than 99%. (Right) Diagrammatic representations of the nucleotide differences between the master sequence and the sequences obtained by limit dilution (LD) from the five individuals. The master and LD sequences from each subject are represented by horizontal black lines. The horizontal axis indicates nucleotide positions in the C2-V5 region. Nucleotide differences between the master and LD sequences are indicated by ticks, with the color of the tick indicating the base present (A in green, C in blue, G in orange, and T in red). Mismatch analyses were performed using the highlighter tool, available at https://www.hiv.lanl.gov/content/sequence/HIGHLIGHT/highlighter_top.html.

The complete *env* gene sequence was determined for patients DET 763, DET 887, DET 892, DET 902, and DET 936 in samples at 55, 96, 47, 159, and 108 dpi, respectively. Sequence phylogenetic analysis (C2-V5 region) of the virus circulating in plasma revealed a low viral population diversity consistent with the short time elapsed since infection. However, it should be noted that the diversity observed in individuals who demonstrated greater neutralization breadth, DET 887 and DET 936, was greater, and the differences cannot be attributed to time elapsed since infection in each individual (Fig. 2). To further investigate this point, sequencing of additional DET 887 amplicons would be required, but, unfortunately, no more DET 887 amplicons were obtained due to RNA sample availability.

For sequence characterization, the major viral population and phylogenetic associations were determined by neighbor joining with 43 amino acid sequences from reference viruses. All envelope sequences branched together with reference subtype B sequences, and the same phylogenetic associations were observed at reverse transcriptase (RT) and protease (data not shown). The length of the variable regions and the potential N-linked glycosylation (PNG) positions were defined on the basis of the HXB2 reference sequence, and data from the patients selected in this study have been compared to 440 subtype B sequences from the Los Alamos database (https://www.hiv.lanl.gov/), with 71 sequences >40 and <160 dpi and 369 sequences ≥2 years postinfection (Table 4).

**TABLE 4:** Variable loop length (in aa) and potential N-glycosylation sites of viral *env* genes amplified from selected recently infected patients with broadly neutralizing responses

Patient or sequence	V1 length	V2 length	V1-V2 length	V3 length	V4 length	V5 length	gp160 length	NXS/PNGS^*a*^	NXS/NXT^*b*^	N332^*c*^
DET 763 (55 dpi)	24	47	71	35	33	12	860	0.39	0.67	YES
DET 887 (96 dpi)	25	42	67	35	29	11	849	0.25	0.33	NO
DET 892 (47 dpi)	31	40	71	35	27	11	854	0.34	0.53	YES
DET 902 (108 dpi)	31	43	74	35	30	12	859	0.28	0.39	NO
DET 936 (159 dpi)	37	45	82	34	36	12	871	0.27	0.41	NO
HIV *env* database sequence (40 to 160 dpi)^*d*^	28.2 ± 5.4	41.3 ± 3.6	69.4 ± 6.4	34.9 ± 0.3	32.6 ± 3.2	11.7 ± 2,2	857.5 ± 7.8	0.33	0.52	ND
HIV *env* database seq (≥2 yrs postinfection)^*e*^	29.8 ± 5.1	41.1 ± 4.3	70.9 ± 6.1	34.7 ± 1.5	31.7 ± 3.5	11.4 ± 1.6	856.7 ± 9.7	0.33	0.51	ND

a NXS potential N-glycosylation sites/all potential N-glycosylation sites (PNGS) ratios.

b NXS potential N-glycosylation sites/NXT potential N-glycosylation sites ratios.

c Asp at position 332 in gp160.

d Calculated out of 71 subtype B sequences from Los Alamos database (http://www.hiv.lanl.gov).

e Calculated out of 369 subtype B sequences from Los Alamos database (http://www.hiv.lanl.gov).

When analyzing the length of the variable loops, the only relevant difference we have observed was in the length of the V1-V2 loop in DET 936, which was 82 amino acids (aa) long and is 12 aa longer than the average length observed for the 440 subtype B sequences analyzed in both recent and chronic infection. This observation is in contrast with what has been previously reported for transmitter/founder viruses and viruses from recent infection (22, 23).

Next, the potential N-linked glycosylation sites (PNGS) were analyzed in our envelopes since lower asparagine-X-serine (NXS)/PNGS and lower NXS/asparagine-X-threonine (NXT) ratios have been associated with a higher probability of broadly neutralizing responses induction (24, 25). Three of the envelopes included in the present study (DET 887, DET 902, and DET 936) showed NXS/PNGS and NXS/NXT ratios lower than the average values observed for individuals in both chronic and recent infection (0.25, 0.28, and 0.27 for NXS/PNGS ratios; 0.33, 0.39, and 0.41 for NXS/NXT ratios, respectively) (Table 4).

**FIG 3:**
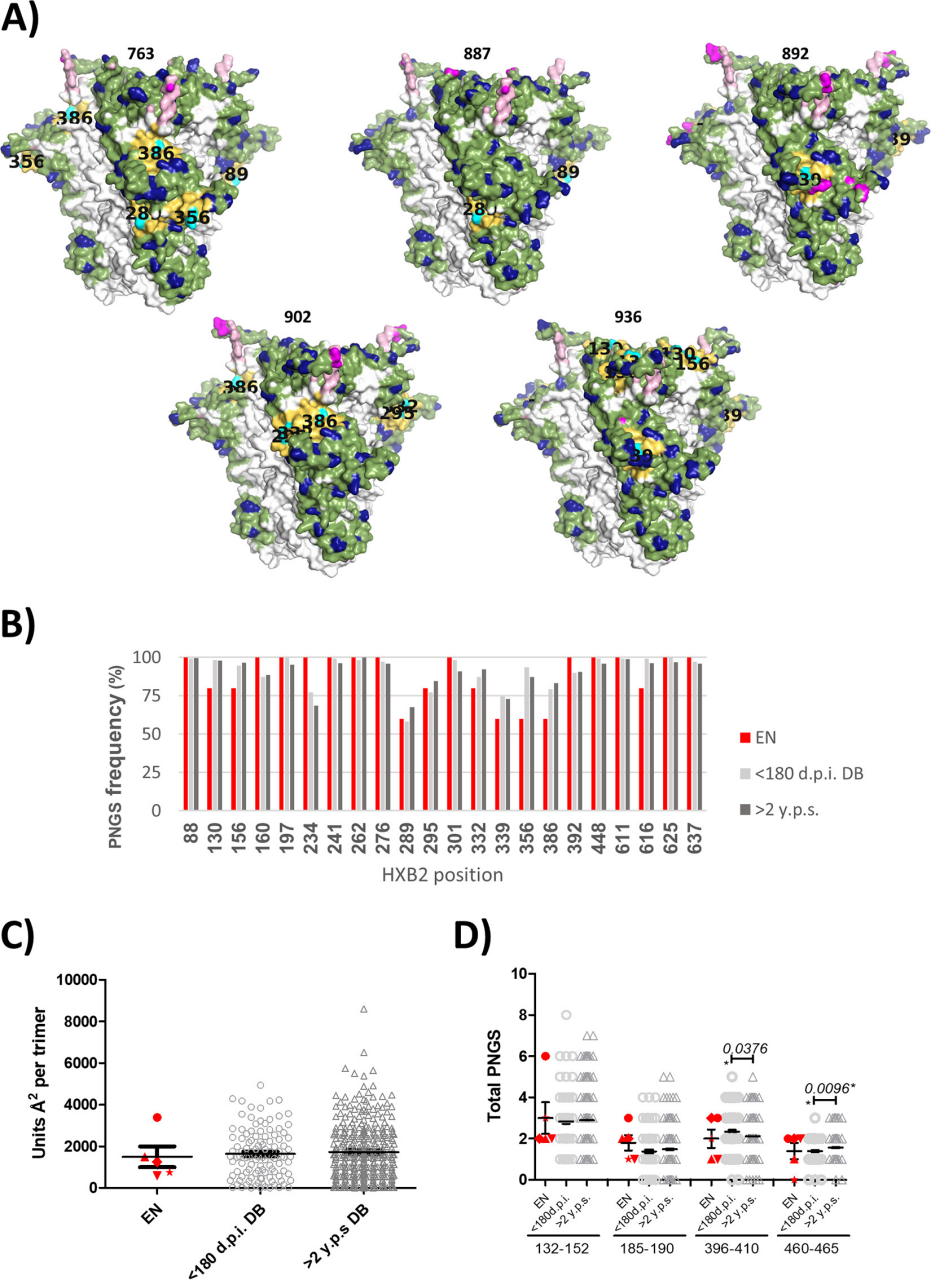
Glycan hole area prediction in patients with early neutralizer individuals (EN). (A) Mapping of the absent hole-causing PNGS on the predicted glycan shields (side view). The PNGS are shown in cyan with their position labeled in black on the structure, and their 10-Å neighbors are shown in yellow. (B) Frequency of PNGS at common M-group positions in EN individuals (red), in 110 individuals within the first 180 days of infection (light gray), and in 324 subtype B viruses collected more than 2 years after seroconversion (dark gray). (C) Predicted total glycan hole area (using the >50% M-group conserved glycan shield as baseline; Å^2^ per trimer) (27) in the three patient groups: EN in red (763, circle; 887, triangle; 892, star; 902, diamond; and 936, inverted triangle); <180 dpi (open light gray circles), and >2 years post-seroconversion (y.p.s.) (open dark gray triangles). (D) PNGS located in the hypervariable loops (V1, 132 to 152; V2, 185 to 190; V4, 396 to 410; and V5, 460 to 465; positions numbered according to HXB2) for viruses from the three groups indicated above.

Our analysis also showed that viruses from individuals DET 887, 902, and 936 missed a potential glycosylation site at position 332. The absence of glycosylation at this position has been associated with the induction of cross-neutralizing responses (26). On the other hand, the accelerated development of broadly neutralizing responses has been associated with transmitting viruses with more intact glycan shields (27). To study this feature, we compared the integrity of the glycan shield in these viruses to 110 subtype B viruses from individuals within the first 180 days of HIV infection and 324 subtype B viruses more than 2 years after seroconversion. The predicted total glycan hole area, excluding the hypervariable loops, was determined. No significant differences were observed in predicted total glycan hole area in individuals with less than 180 days of infection (range, 0.0 to 4938.9 Å) compared to viruses present after 2 years of seroconversion (range, 0.0 to 8597.2 Å) (Fig. 3C). However, among the early neutralizers, we observed that the average size of the glycan hole was smaller in the envelopes from individuals DET 887, DET 892, DET 902, and DET 936. Envelope from individual DET 763, however, showed a glycan hole that was 2-fold larger (Fig. 3A and C). Next, we analyzed the frequency of PNGS that are common in group M in all three patient groups. The frequency of PNGS at common M group positions for recent and chronic viruses are shown in Fig. 3B. No differences in PNGS frequency were observed at the indicated positions between viruses with recent and chronic infection. Early neutralizer viruses also showed similar frequencies at those positions (Fig. 3B).

Next, we studied the contribution of PNGS located in hypervariable loops to the glycan shield in all three groups of individuals. The number of PNGS at each hypervariable loop for viruses at recent and chronic infection are shown in Fig. 3D. No differences were observed between the number of PGNSs in V1 and V2 hypervariable regions in patients with recent and chronic infection. However, significant differences in the number of PNGS in V4 and V5 hypervariable regions were observed, as individuals with recent infection had more PNGS in the V4 hypervariable region and less PNGS in the V5 hypervariable region than viruses from chronic infection time points (Fig. 2D). When studying the contribution of PNGS located in hypervariable loops to the glycan shield in early neutralizer viruses, no significant differences were observed between these viruses and viruses from both recent and chronic infection for any of the hypervariable regions, but this observation may be due to the small number of early neutralizer viruses included in the study. In the case of 763 Env, we observed more PNGS in hypervariable loops than the average of the three comparison groups, especially in the V1 loop. This difference could compensate for the lower integrity of the glycan shield in the rest of the protein (Fig. 3A and C). The present analysis revealed no association between glycan shield integrity and the ability to induce broadly neutralizing responses in recent infection.

### Generation and characterization of HIV-1-Gag VLPs.

Based on their sera-neutralizing capacity, their low NXS/NXT ratios, and the absence of N332, viral envelopes present in DET 887 and 936 early neutralizers were selected to design immunogens based on HIV-1 envelopes incorporated into VLPs.

**FIG 4:**
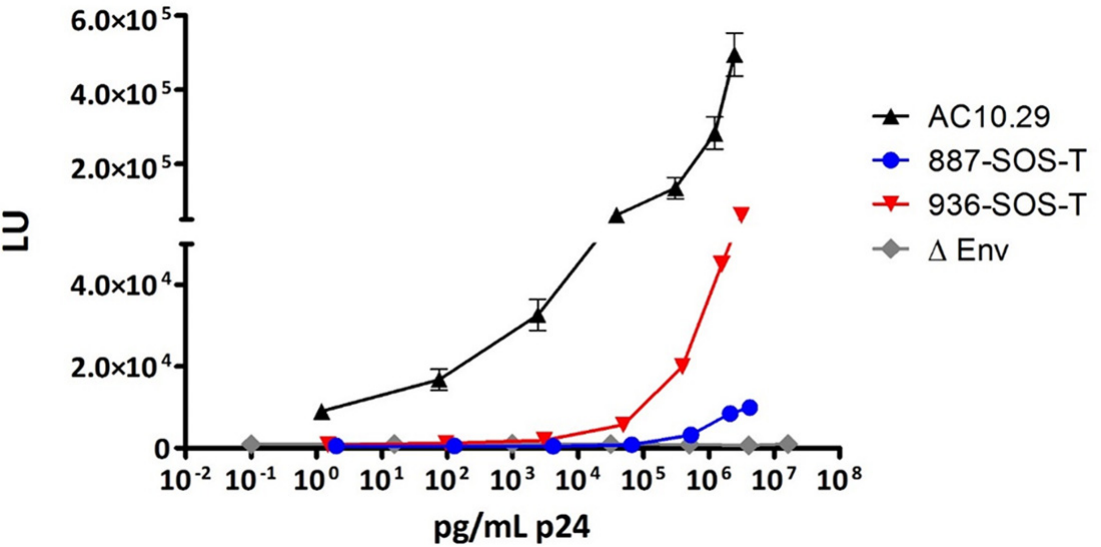
Comparative effect of SOS-T mutations in EN pseudoviruses. Virus stocks were obtained from cotransfection of 887-SOS-T and 936-SOS-T expression plasmids with an *env-*deficient HIV backbone (pNL4-3ΔenvFL) on HEK-293T cells, normalized for the amount of p24, and used to infect TZM-bl cells in triplicate. At 48 h postinfection, luciferase activity was evaluated and expressed as luciferase units (LU).

To generate HIV-1-Gag VLPs from the selected envelopes, their sequences were modified to improve trimer stability by incorporation of SOS and T mutations (A501C plus T605C and I/L535M plus L543Q, respectively) (28–31). To determine the functionality of these envelopes, we generated pseudotyped viruses from these envelopes, and their infectiveness in TZM-bl cells was evaluated. Viruses 887-SOS-T and 936-SOS-T showed reduced infectivity levels compared to the infectivity of a pseudotyped virus generated with the reference strain AC10.29 under the same conditions (35-fold reduced for 887-SOS-T and 8-fold reduced for 936-SOS-T) (Fig. 4). This remarkable loss of infectivity could be attributed to the presence of SOS-T mutations that prevent dissociation of gp120 from gp41 (32). Despite their limited replication capacity, both viruses proved to be infectious, confirming the ability of 887-SOS-T and 936-SOS-T envelopes to generate functional trimers (Fig. 4).

**FIG 5:**
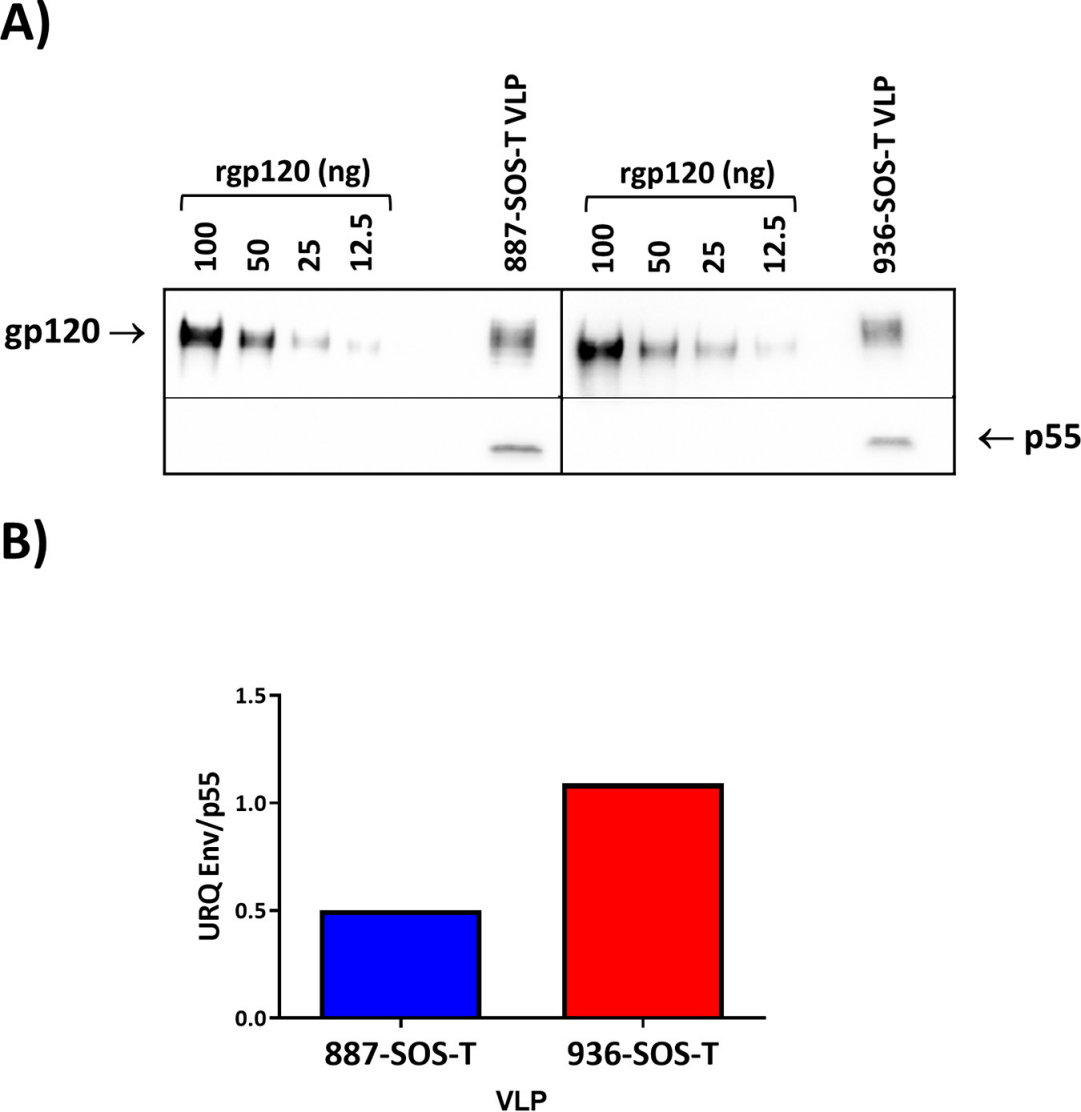
Env incorporation per VLP. VLPs were generated in 293F cells, and Env incorporation was analyzed by Western blotting in SDS-PAGE gels as indicated in Materials and Methods. (A) VLPs gp120 quantification with a rgp120 (Bal) standard curve. gp120 and p55 were detected with 447-52D and p24 (24-4) antibodies, respectively. (B) Relative Env (gp120) incorporation into VLPs. The ratios of gp120 to p55 were calculated by using phosphorimaging analysis.

**FIG 6:**
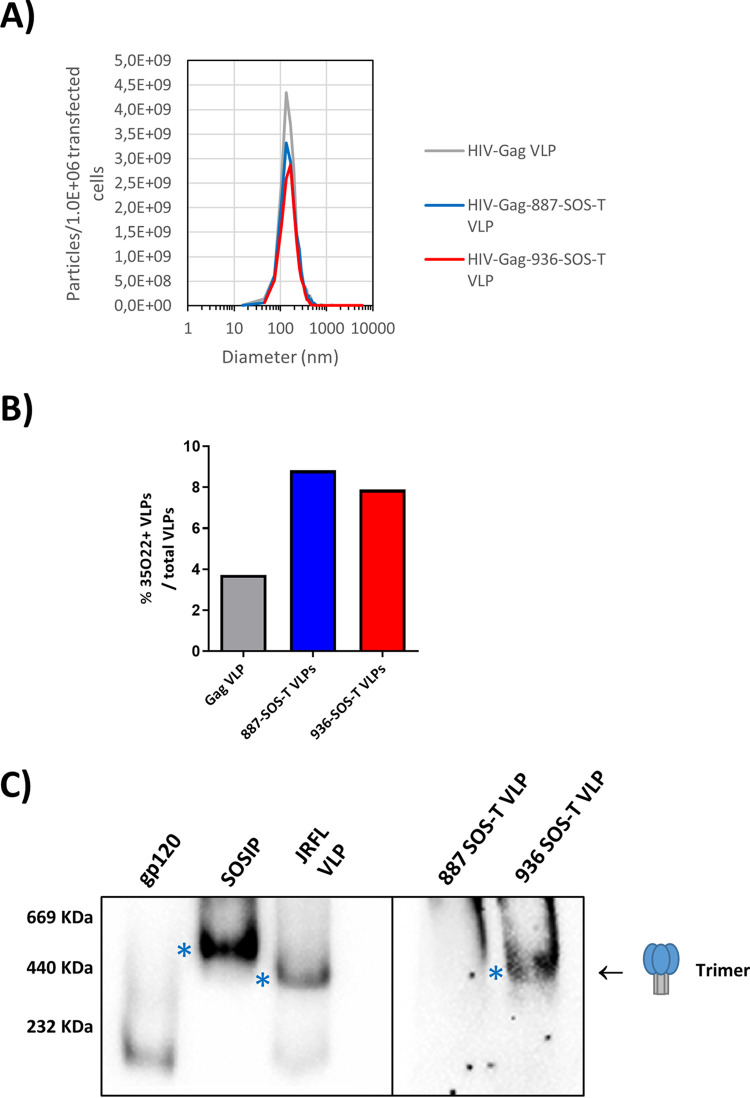
Env-VLPs characterization. (A) Particle size distribution analyzed by NTA. (B) Percentage of VLPs that incorporated envelopes in a closed trimeric conformation determined by NTA after immunolabeling with DY-405-conjugated 35022 antibody. (C) Trimer detection analyzed by Western blotting in BN-PAGE gels. An AC10.29 rgp120 (gp120 monomer), a 96ZM651 SOSIP (trimer), and a JRFL-VLP (VLP-incorporated trimer) were used as controls. Trimers were detected with an α-gp120 and α-gp41 antibody cocktail. Trimers are indicated with asterisks.

HIV-1 Gag virus-like particles with no envelope and carrying both SOS-T variants were generated, and VLP Env incorporation was evaluated by Western blotting (Fig. 5). VLP incorporation was twice as efficient for the 887-SOS-T variant than 936-SOS-T, and trimers could only be detected in 936-SOS-T VLPs (Fig. 6C). Particle size distribution was analyzed by nanoparticle tracking analysis (NTA). VLPs containing dGag, dGag-887 Env, and dGag-936 Env showed a monodisperse distribution and similar size distribution, with average diameters of 161.3, 165.0, and 164.2 nm, respectively. The production yield was similar for both Env-VLPs (3.0 × 10^9^ and 3.0 × 10^6^ transfected cells) and somewhat higher for VLPs carrying no Env (4.5 × 10^9^ and 4.5 × 10^6^ transfected cells) (Fig. 6A). The percentage of VLPs that incorporated envelopes in a closed trimeric conformation on their surface was also determined by NTA after labeling VLPs with the 35022 trimer-specific antibody. The percentage of 35022-positive VLPs was below 10% (Fig. 6B). Although the level of trimer incorporation is low, it is not surprising, considering that 887 and 936 envelopes have few modifications compared to the original patient sequences with an untruncated transmembrane protein, and the estimated number of spikes per wild type HIV virion is 7 to 14 (33).

### Comparative antigenicity of 887-SOS-T and 936-SOS-T HIV-1-Gag VLPs.

VLPs were tested against previously described bNAbs that are specific for prefusion-native trimers (PG16, PGT145, VRC01, PGT151, and 35022), the CHO-dependent bNAb 2G12, and a CD4-inducible nonneutralizing antibody (17b). As a control, we also examined VLPs that bear no Env.

**FIG 7:**
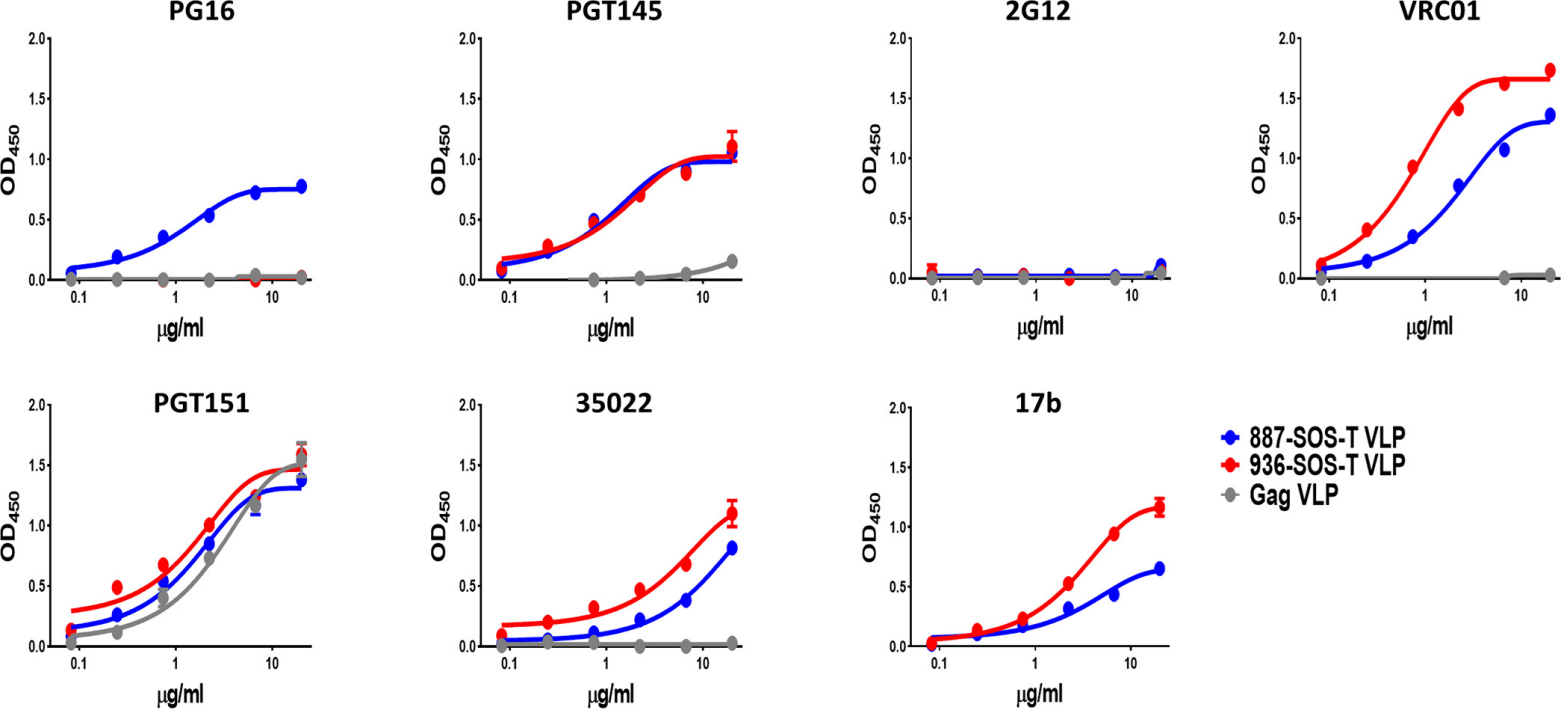
VLPs antigenicity analysis. Antigenicity of VLPs formulated Envs was evaluated by ELISA with a panel of monoclonal antibodies with different specificities. VLPs were normalized by gp120 and immobilized in ELISA plates. VLPs with no envelope incorporated were used as specificity controls (Gag VLP).

Most of the antibodies tested bound specifically to Env-VLPs as confirmed by their general lack of reactivity with VLPs carrying no envelope. However, PGT151 antibody showed strong nonspecific binding, suggesting a possible cross-reactivity with VLP membrane components (Fig. 7). PGT151 binding to glycan complexes in several cell types has been described previously (34). The 887 and 936 VLPs incorporated envelopes that specifically recognized bNAbs PGT145, VRC01, and 35022. Binding to PGT145 was similar for both envelopes. The 936 VLP-incorporated envelope showed superior binding to VRC01 and 35022 antibodies than 887 VLP (Fig. 7). In contrast, bNAb PG16 showed binding to 887 but not to the 936 VLP-associated envelope.

The 17b antibody bound both envelopes, suggesting the coexistence of Env-VLPs with abnormal conformation and envelopes with a native closed trimeric conformation on the VLP surface.

In light of these results, we can infer that Env-VLPs from individuals with bNA in recent infection incorporate a substantial number of trimers in native conformation. However, these VLPs also have defective trimers on their surface that could interfere with the induction of broad-spectrum neutralizing responses.

### Immunogenicity of 887-SOS-T and 936-SOS-T HIV-1-Gag VLPs.

**FIG 8:**
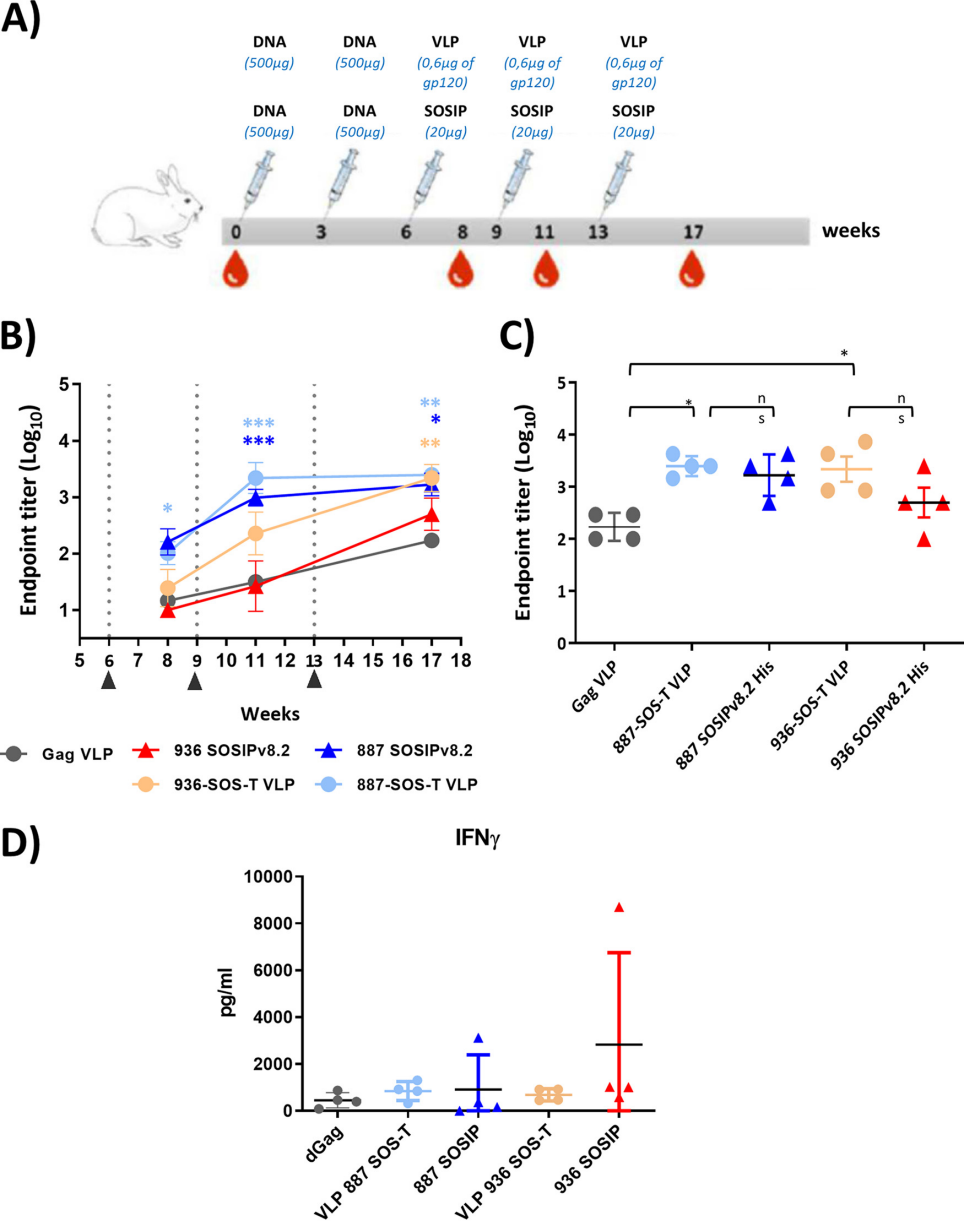
Characterization of immune responses induced in rabbits immunized with 887-SOS-T and 936-SOS-T VLPs and the corresponding SOSIPs. (A) Immunization protocol. Animals were immunized with two DNA primes and 3 VLP or SOSIP boosts at 3-week intervals. Two weeks after the last boost, rabbits were exsanguinated. (B) ELISA sera endpoint titers against Bal rgp120 during the immunization process (*, *P* < 0.05; **, *P* < 0.01; ***, *P* < 0.001; two-way ANOVA Bonferroni posttest compared to the VLP Gag immunized group). VLPs or SOSIPs boosts are indicated. (C) ELISA sera endpoint titers against Bal rgp120 at exsanguination, week 17 (n.s., not significant; *, *P* < 0.05; Mann-Whitney U test). (D) IFN-γ sera levels at exsanguination, week 17.

To further evaluate the humoral response induced by immunogens based on the selected viral envelopes, rabbits were immunized with HIV-1-Gag VLPs. In order to compare immunogenicity of Env-VLPs with the corresponding stabilized soluble trimers, additional groups of rabbits were immunized with SOSIPs generated from the same HIV-1 Envs generously provided by Rogier Sanders (887 SOSIPv8 and 936 SOSIPv8). Rabbits were immunized with a DNA prime/protein boost protocol considering that previous reports have shown DNA priming steps to strongly favor the development of both humoral and cellular responses upon subsequent protein boost (35). Accordingly, animals were primed with two DNA inoculations followed by three VLP or SOSIP booster vaccinations (G_2_, 887-SOS-T VLPs; G_3_, 887 SOSIPsv8; G_4_, 936-SOS-T VLPs; and G_4_, 936 SOSIPsv8) (Fig. 8A). An additional group of rabbits was immunized with VLPs carrying no envelope (G_1_, Gag VLPs). Considering that the origin of the immunogens designed in this study was the viral envelope sequence from individuals with broadly neutralizing activity in recent infection, we decided to use a quick immunization protocol (105 days) in order to recapitulate what happened during natural infection.

All immunized rabbits developed responses to heterologous, monomeric rgp120 (Bal) (Fig. 8B) that was more potent in rabbits immunized with 887 VLPs and SOSIPs. In fact, both groups of rabbits reached the maximum anti-gp120 endpoint titer (5 × 10^−3^) at week 11. In contrast, rabbits immunized with 936 VLPs did not reach a 10^−3^ titer until week 17, and in the case of rabbits immunized with 936 SOSIPv8.s, the maximum titer achieved was 10 times lower. When comparing the antigp120 antibody titers on the day of final bleeding (week 17), no significant differences between the groups immunized with VLPs and SOSIPS were observed (G_2_ versus G_3_ and G_4_ versus G_5_) (Fig. 8C).

Remarkably, rabbits immunized with HIV-1-Gag VLPs with no HIV-1 envelope showed some gp120 recognition that reached 2 × 10^−2^ titer at week 17. Given that rgp120 and VLPs were both generated in similar cell lines, binding to gp120 observed in enzyme-linked immunosorbent assay (ELISA) could be attributed to the recognition of cellular protein traces present in the rgp120 preparation. Nevertheless, rabbits immunized with 887 and 936 VLPs had significantly superior anti-gp120 titers than rabbits immunized with HIV-1-Gag VLPs.

On the other hand, we investigated T cell responses in immunized rabbits by quantifying interferon gamma (IFN-γ) in serum, but no significant differences among groups were found (Fig. 8D).

### Autologous VLP and SOSIP recognition.

**FIG 9:**
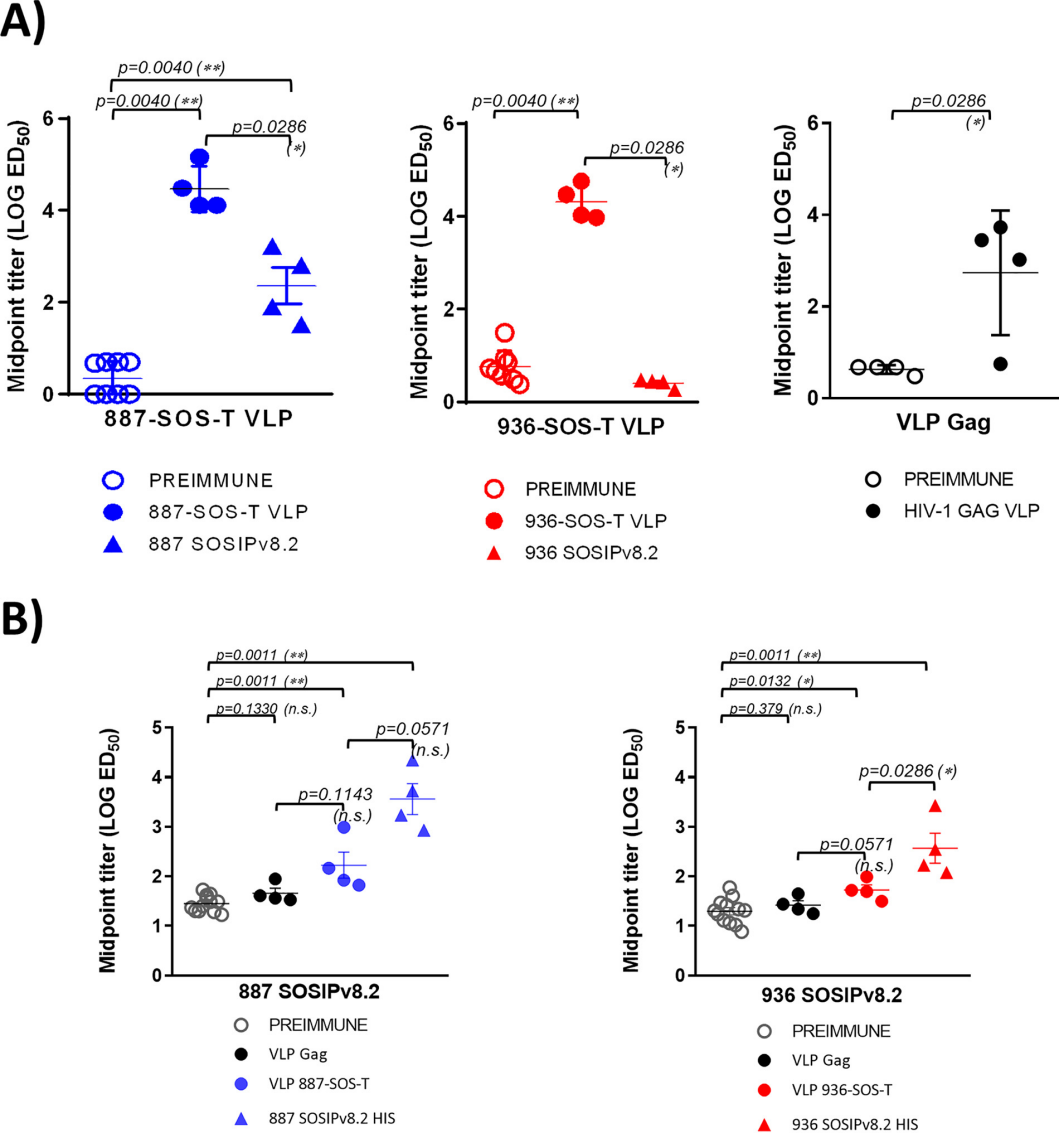
Characterization of VLP and SOSIP responses induced in rabbits immunized with the autologous corresponding VLPs and SOSIPs. (A) VLP reactivity in sera from rabbits immunized with autologous VLPs or SOSIPs at exsanguination and the corresponding preimmune sera. 887-SOS-T and 936-SOS-T VLPs were normalized by gp120 and immobilized in ELISA plates, and sera reactivity was determined. (B) SOSIP reactivity in sera from rabbits immunized with autologous VLPs or SOSIPs at exsanguination and the corresponding preimmune sera. Histidine-tagged SOSIPS v8.2 were immobilized in NTA-Ni ELISA plates, and sera reactivity was determined. Reactivity in sera from rabbits immunized with VLPs and SOSIPs is indicated with closed circles and closed triangles, respectively (887-SOS-T in blue, 936-SOS-T in red and Gag VLP in black). Reactivity of preimmune sera is indicated with open gray circles.

The 887 and 936 VLPs were recognized by sera from rabbits immunized with autologous VLPs (Fig. 9A). It is worth noting that sera from rabbits immunized with VLPs without envelope also recognized the corresponding VLPs, suggesting the presence of antibodies directed against cellular components present in VLPs (Fig. 9A). Sera from rabbits immunized with 887 SOSIP recognized 887 VLPs. The presence of Env-VLP cross-recognition in 887 SOSIP-immunized rabbit sera suggests that 887-VLPs incorporate autologous trimers in a conformation similar to that presented by the corresponding SOSIPs. However, sera from rabbits immunized with 936 SOSIPs were unable to recognize autologous VLPs (Fig. 9A).

Conversely, 887 and 936 SOSIPs were recognized by sera from rabbits immunized with both 887 or 936 immunogens (Fig. 9B), with greater recognition of the autologous sequence. However, it should be noted that sera from rabbits immunized with VLPs with no envelope recognize 887 and 936 SOSIPs, probably due to the presence of traces of cellular components from the producer cells present in SOSIP stocks. These results suggest that, despite that some antibodies recognized cellular components present in VLPs, rabbits immunized with Env-VLPs were also able to recognize autologous trimers in native conformation.

Before testing rabbit sera for neutralization, sera from VLP-immunized rabbits were preadsorbed with 293F cells to deplete sera from non-Env antibodies that could render nonspecific neutralization. IgGs were purified from preadsorbed rabbit sera and tested against the minipanel of recombinant viruses described before at the highest IgG concentration that showed no toxicity and no neutralization of the VSV-Env-pseudotyped control. No neutralizing activity against any of the viruses tested was observed (Table 5). Similarly, no neutralization was observed when purified IgGs were tested with autologous pseudotyped 887-SOS-T and 936-SOS-T viruses (Table 5).

**TABLE 5:** Neutralization of sera-purified IgGs from immunized rabbits

Immunogen	Rabbit	IC50 (μg/ml) of:							
		VI 191, subtype A, tier 2	AC10.029, subtype B, tier 2	NL4-3, subtype B, tier 1A	92BR025, subtype C, tier 1B	92UG024, subtype D, tier 2	CM244, subtype AE, tier 2	887-SOS-T pseudovirus^*a*^	936-SOS-T pseudovirus
887-SOS-T vLP	1769	>500	>500	>500	>500	>500	>500	>200	ND
	1775	>500	>500	>500	>500	>500	>500	>200	ND
	1776	>100	>100	>100	>100	>100	>100	>200	ND
	1777	>100	>100	>100	>100	>100	>100	>200	ND
887 SOSIP	1768	>500	>500	>500	>500	>500	>500	>200	ND
	1772	>500	>500	>500	>500	>500	>500	>200	ND
	1774	>100	>100	>100	>100	>100	>100	>200	ND
	1778	>100	>100	>100	>100	>100	>100	>200	ND
936-SOS-T vLP	1783	>200	>200	>200	>200	>200	>200	ND	>500
	1784	>200	>200	>200	>200	>200	>200	ND	>500
	1785	>200	>200	>200	>200	>200	>200	ND	>500
	1786	>200	>200	>200	>200	>200	>200	ND	>500
936 SOSIP	1787	>200	>200	>200	>200	>200	>200	ND	>500
	1788	>200	>200	>200	>200	>200	>200	ND	>500
	1789	>200	>200	>200	>200	>200	>200	ND	>500
	1790	>200	>200	>200	>200	>200	>200	ND	>500

*^a^*ND, not determined.

## DISCUSSION

From a historical point of view, vaccinology has been successful in preventing infections of pathogens that expressed invariable antigens and which could be neutralized efficiently by antibodies. However, HIV-1, as a pathogen with high sequence variability, has presented a major challenge for vaccine researchers, requiring new approaches and vaccine strategies (36).

It is still generally accepted that a minimum of 2 years of infection are needed to generate bNAbs since long periods of somatic maturation in the presence of the antigen may be required to generate such antibodies. If this hypothesis is true, inducing bNAbs by a preventive vaccine may require complex and long vaccination strategies.

Our group published, in 2016, a study identifying broadly neutralizing responses in HIV-infected individuals within the first 6 months of infection (21). Based on this cohort of individuals, the present study was focused on the detailed study of viral envelope proteins of individuals whose bNAbs were induced the earliest in infection. Specifically, from a cohort of 88 individuals within the first 100 days of infection, we selected 5 that were able to neutralize tier 2 viruses and cross-neutralize at least 3 viral subtypes (DET 763, 892, 902, 887, and 936) (Table 3). Within these individuals, DET 763 showed the earliest neutralization breadth with serum from day 28 of infection that neutralized viruses from 3 subtypes out of a tested minipanel. Two other individuals, DET 887 and DET 936, showed the highest activity, neutralizing viruses of 4 subtypes out of the minipanel with an ID_50_ of 100 at 96 and 82 days postinfection, respectively.

These results suggest that, under certain circumstances, the immune system is able to generate bNAbs against different HIV-1 subtypes in a short period of time. In a first study, published in 2011, an infected individual was identified who, at 9.8 months postinfection, was able to neutralize 3 different viral subtypes. The virus isolated from this individual at 3.2 months postinfection had very long V1 regions in the gp160 sequence compared to the variants of other chronic elite neutralizers (37). In 2014, two studies were published identifying individuals who developed bNAbs around 1 year of infection (38, 39). In the same year, another report showed that bNAbs can be detected in HIV-1-infected infants at birth (40). Seven of these studied infants (25%) were able to neutralize two or more viruses from four different subtypes in an average time of 20 months postinfection. This study demonstrated that bNAbs can develop within the first year of life and suggested that, despite having an immature immune system, infants maintain functional B cells capable of mounting broadly neutralizing responses (40). This observation is consistent with what has been described for mature T cell responses that have been detected even in umbilical cord (41). In addition, viral *env* sequences from one of these infants were determined, and immunogens based on these sequences were developed. One of these sequences, BG505, is currently the structural reference of the native Env trimer used by numerous researchers, and immunogenicity studies of BG505 SOSIPs have shown that this immunogen is capable of inducing tier 2 autologous responses in rabbits and macaques (42–44).

Plasma virus sequences from individuals with broadly neutralizing responses in recent infection were analyzed to further investigate the viral factors associated with the induction of broadly neutralizing responses in recent infection. Overall, a low viral quasispecies diversity was observed. Based on this evidence, we inferred that, in the individuals included in this study, neutralization breadth could not be attributed to superinfection events, as previously suggested by some studies (45, 46).

We have also observed that individuals with the highest range of neutralizing Ab response (DET 887, 902, and 936) had the lowest NXS/NXT ratios compared with the ratios found in Env sequences of individuals at 40 to 160 days postinfection (Table 4). These findings are in line with other studies in which lower NXS/PNGS and NXS/NXT ratios were correlated with an improved neutralization breadth (25).

Another genetic characteristic, which has been correlated with the increased induction of bNAbs in chronically infected individuals, is the length of the Env variable loops, especially the V1-V2 loop (47). In contrast, in the case of viruses from recent infection, a shorter V1 length has been correlated with a higher subsequent induction of bNAbs (24). The main limitation of our study in inferring correlations conclusively on this aspect is due to the loop length variability and the small number of samples (Table 4).

Taking into account all the data and despite the limitations due to the small number of envelopes analyzed, our data suggest that the induction of broadly neutralizing responses in recent infection is associated with a lower NXS/NXT ratio, the absence of N332, and the presence of N334. In order to verify this hypothesis, it will be necessary to analyze a greater number of viral Envs from individuals with wide neutralization breadth in recent infection.

On the other hand, in some of the viral envelopes analyzed, we observed characteristics previously associated with transmitted founder viruses (T/F). Specifically, 887 has a histidine at position 12 of the signal peptide. This has been associated with the traffic of gp160 to the RER, Env incorporation into viral particles, and viral infectivity (22). The 887 and 892 Envs also lacked threonine at position 315, which resulted in the loss of a glycosylation site. This is a pattern that has previously been observed in individuals with recent infection (48). Another feature that has been associated with induction of broadly neutralizing responses during HIV-1 infection is the presence of an intact glycan shield in the transmitted virus (27). In the present study, we found no significant differences when comparing the integrity of the glycan shield or the frequency of PNGS in viruses from individuals with broadly neutralizing responses in recent infection, viruses within the first 180 days of infection, and viruses in samples drawn more than 2 years after seroconversion. However, we cannot determine whether differences were not observed due to the small number of early neutralizers analyzed or because the mechanism of neutralization breadth induction is different in these individuals.

Based on all the characterization of the studied envelopes and their greater neutralization breadth across at least 4 viral subtypes, we selected viral envelopes from DET 887 and DET 936 for immunogen design. Although many optimizations have been developed in previous studies in order to improve Env expression, its stability, or its antigenicity, we decided to minimize the number of additional mutations introduced in order to keep the spike conformation as close as possible to the one which presumably induced broadly neutralizing responses *in vivo*.

VLPs with 887 and 936 Envs were homogeneous, with average diameters of 161 to 165 nm and at least 10% of the total incorporated trimers. Furthermore, VLPs’ antigenicity study confirmed that both VLPs incorporated trimers in native closed conformation, as evidenced by their ability to bind antibodies PGT145, 35022, and PG16, the latter only in 887-VLP. On the other hand, the presence of misfolded envelopes coexisting with native trimers was evidenced by the binding of both VLPs to the nonneutralizing antibody 17b (Fig. 7). This observation is consistent with the coexistence, in HIV virions, of Envs in closed and open conformation together with misfolded trimers that have been previously reported (49).

When evaluating VLPs’ immunogenicity, we had the opportunity to incorporate soluble stabilized trimers from the corresponding Envs into the study. This allowed us to evaluate the immunogenicity of the two viral envelopes in two platforms, VLP associated and SOSIPs.

All immunogens induced detectable levels of antibodies against heterologous monomeric gp120 (Bal), and no significant differences were observed between VLPs and SOSIP, although, in the case of VLPs, the amount of envelope delivered was 30 times lower (Fig. 8). One of the main limitations that has been associated with the use of VLP-formulated HIV Envs is their low incorporation per VLP, which is 7 to 14 spicules per particle, including misfolded forms (50–52). This feature is also characteristic of HIV and simian immunodeficiency virus (SIV) virions and has been implicated in immune evasion mechanisms (33, 53–55). However, our results indicate that the low envelope incorporation in VLPs might not be a problem considering that, to obtain the same antibody titer, fewer Env molecules are needed if they are VLP incorporated.

Rabbits immunized with both 887-based immunogens developed autologous antibodies against both 887-SOS-T VLPs (Fig. 9A) and 887 SOSIPv8.2 (Fig. 9B), suggesting that antibodies have been induced against native Env trimers, a key feature of bNAbs. In contrast, this cross-recognition was not observed with Env 936, and SOSIP recognition by animals vaccinated with the corresponding VLP was very low.

The immunogenicity of the same envelope, membrane incorporated and in a SOSIP format, has been compared previously in nonhuman primates. These studies did not show major differences in the antibody induction and autologous neutralizing responses between both vaccine prototypes (1, 44). The fact that SOSIPs did not induce autologous neutralizing response in rabbits (Table 5) was unexpected because the previous SOSIP prototype, SOSIPv.7, has been tested on different animal models and demonstrated ability to induce autologous neutralizing response (30). We cannot rule out the possibility that the new mutations incorporated to generate SOSIPsv8.2, in the context of the envelopes selected in this study, have caused conformal changes in the protein that result in a lower ability to induce autologous neutralizing responses.

Alternatively, the absence of autologous neutralization could be due to the immunization protocol chosen in this study in an attempt to mimic the *in vivo* scenario in the infected individual; we delivered all intervals of 3 weeks over a total period of only 17 weeks. Immunization protocols in previous studies with soluble trimers that successfully induced autologous neutralizing responses are much longer, and immunizations were more spaced. In fact, there is evidence confirming that longer intervals between immunizations allow germ line center (GC) response to mature after each administration, generating the hypermutation needed to obtain receptor affinity from B cells by neutralizing epitopes (44). In view of the results of this study, the applied vaccination regimen is presumably not an appropriate protocol for neutralizing responses induction. On the other hand, we cannot rule out that the choice of adjuvant has influenced the results obtained. A recent study showed successful induction of neutralizing responses with a similar approach using granulocyte-macrophage colony-stimulating factor (GM-CSF) as a DNA adjuvant in addition to Adjuplex as a protein adjuvant. In the present study, no adjuvant was used for DNA immunization, and this may have also influenced the results obtained (56).

Given that a small number of individuals have been identified that are capable of inducing broadly neutralizing responses very early in infection, we can infer that induction of such responses by vaccination is feasible. For this reason, we have developed a line of research based on the design of immunogens mimicking the corresponding viral envelopes’ VLP formulated with the purpose of recapitulating, by vaccination, the antigenic stimulation that resulted in antibody induction in these individuals. Both prototypes have demonstrated ability to present envelopes in native trimeric conformation and to induce an antibody response against heterologous gp120 similar to the responses induced by corresponding stabilized soluble trimers.

To progress in the development of this line of immunogens, the identification of a greater number of early neutralizer individuals would be required in order to define the factors associated with induction of a greater breadth of response in the early stages of infection.

## MATERIALS AND METHODS

### Epitope mapping.

### (i) Neutralization assays.

To characterize neutralizing antibodies, neutralization assays with single-round HIV-1 Env pseudovirus infection were performed using a luciferase-based assay in TZM-bl cells. To determine the serum concentration causing a 50% reduction of relative light unit value, 4-fold serial dilutions (1:20 to 1:327,680) were made. The neutralization dose-response curves were fitted by nonlinear regression using a four-parameter hill slope equation. For the mapping of neutralizing antibodies directed to glycan structures in the variable region (V1, V2, and V3), neutralization assays were performed using JRCSF virus with the N160K mutation and the N332A mutation, respectively. For the mapping of NAbs to the MPER of gp41, sera were tested for neutralizing activity against a chimeric HIV-2 virus containing the MPER of HIV-1 (7312.C1) and the parental HIV-2 7312A clone.

### (ii) ELISAs.

Enzyme-linked immunosorbent assays (ELISAs) were performed as previously described (57). ELISA plates were coated with the following probes: the antigenically resurfaced glycoprotein RSC3 containing the CD4bs, the RSC3 Δ371I/P363N mutant, and the RSC3 G367R probe. Sera that showed a loss of reactivity to the RSC3 P363N mutant but reacted to both RSC3 and RSC3 G367R were classified as containing CD4bs antibodies.

### Sequence generation and phylogenic analysis.

Viral RNA was amplified by nested reverse transcriptase PCR (RT-PCR) for the *env* gene, bulk PCR for full-length *env* sequence, and C2-V5 for single-genome amplification. RT-PCR was performed using the one-step RT-PCR kit (Qiagen, Germany). One microliter of the first PCR product was reamplified using Expand high-fidelity enzyme following the manufacturer’s instructions (Thermo Fisher, MA, USA). Full-length Envs were amplified from serial 10-fold dilutions of each RNA template. The amplicons generated from the dilution prior to the last positive PCR were selected for sequencing in order to avoid bottlenecking.

Nucleotide sequences were determined by the BigDye Terminator cycle sequencing kit (Applied Biosystems, Thermo Fisher Corporation, MA, USA) in an ABI 3730 sequencer (Applied Biosystems, Thermo Fisher, MA, USA) We used SeqMan Pro v12.3.1 (DNASTAR) and BioEdit Sequence Alignment Editor (v7.0.5.3) for assembling and hand editing. Nucleotide sequences were aligned, and the evolutionary history was inferred using the neighbor-joining method using MEGA program (vX.10). The evolutionary distances were computed using the maximum composite likelihood method. In addition to individual phylogenetic trees, we also built a tree with all participants, and no contamination was observed (data not shown).

### Glycan hole area prediction.

Envelope sequences from 110 subtype B viruses from patients within the first 180 days of infection and 324 subtype B viruses more than 2 years after seroconversion were obtained from the Los Alamos National Laboratories (LANL) HIV sequence database (https://www.hiv.lanl.gov/components/sequence/HIV/search/search.html). Glycan shield prediction for the HIV Env sequences was calculated with the Glycan Shield Mapping tool at LANL HIV sequence database (https://www.hiv.lanl.gov/content/sequence/GLYSHIELDMAP/glyshieldmap.html) (27). This tool predicts the total glycan hole area using the >50% M group conserved glycan shield as baseline and the potential N-linked glycosylation sites (PNGS), which are commonly found in HIV-1 group M Envs but absent from the given Env, that gave rise to the glycan holes (positions numbered according to HXB2 numbering, 88, 130, 156, 160, 197, 234, 241, 262, 276, 289, 295, 301, 332, 356, 386, 392, 448, 611, 616, 625, and 637). PNGS that cause the holes in the hypervariable loops (V1, 132 to 152; V2, 185 to 190; V4, 396 to 410; and V5, 460 to 465; HXB2 numbering and V3 does not have a hypervariable region) are not included in the analysis made with this tool because their high variation in sequence, length, and structural orientation make predictions unreliable.

### Pseudotyped virus stocks.

Env-pseudotyped viruses were generated by cotransfection of codon-optimized pcDNA3.1-887-SOS-T or pcDNA3.1-936-SOS-T envelope expression plasmids provided by GeneArt Gene Synthesis (Thermo Fisher Scientific, Waltham, MA, USA) and an *env*-deficient HIV backbone, pNL4-3ΔenvFL (58) on HEK-293T, as previously described (59).

### Virus-like particle generation.

For VLP generation, a full-length HIV-1 Gag sequence previously optimized as a T cell immunogen was used (dGag) (20). HIV-1-Gag VLPs were produced by transient transfection with the FreeStyle 293 expression system (Invitrogen, Carlsbad, CA, USA) cotransfecting with a pcDNA-dGag plasmid and codon-optimized pcDNA3.1-887 or pcDNA3.1-936 envelope expression plasmids provided by GeneArt Gene Synthesis (Thermo Fisher Scientific, Waltham, MA, USA). For transfection, 1 μg DNA/10^6^cells of each dGag, Env, and furin expression plasmids were used. Different molecular ratios of dGag/Env/furine expression plasmids were tested, and 3.5:1:0.3 and 11.5:1:0.3 ratios were chosen for 887 and 936 VLPs, respectively, in order to obtain similar Env incorporation in both VLPs. At 48 h posttransfection, VLPs were harvested by ultracentrifugation (60). First, VLP supernatants were clarified through two rounds of centrifugation at 800 × *g* for 5 min. Afterward, the supernatants were ultracentrifugated at 50,000 × *g* for 30 min. Pellets were resuspended in phosphate-buffered saline (PBS) and ultracentrifuged once more at 160,000 × *g* for 10 min and resuspended in trehalose 15%-PBS to preserve VLP integrity and stored at −80°C (61).

### Nanoparticle tracking analysis.

Size distribution and particle concentration were analyzed by measuring the rate of Brownian motion using a ZetaView system (Particle Metrix Gmb, Meerbusch, Germany). Samples were serially diluted in particle-free PBS to reach a suitable particle concentration (60 to 100 particles per video frame) for analysis. Videos (60 s) of three dilution steps for each sample were captured at room temperature. The videos were analyzed and evaluated by NTA 2.0 software. The camera level was adjusted manually, and optimized analysis parameters were kept constant during all measurements. The particle number was evaluated for particles with diameters between 10 and 10,000 nm. VLPs were also probed with DY-405 conjugated 35022 MAb, and the percentage of 35,022 positive particles was determined by NTA at the Nanovex Biotechnologies nanoparticle characterization service (http://www.nanovexbiotech.com/services/#characterization).

### Western blotting.

Env incorporation per VLP was analyzed by Western blotting in SDS-PAGE gels (Fig. 5). Identical quantities of p55 were mixed with Laemmli buffer supplemented with 5% β-mercaptoethanol, boiled for 10 min, and kept on ice. Env expression in 293F cells was also analyzed by Western blotting after lysing with a cell lysis buffer (Cell Signaling Technology, Danvers, MA, USA) and boiled for 10 min. Samples were then electrophoresed at 160 V for 90 min through a 4% to 12% polyacrylamide-sodium dodecyl sulfate gradient gel (GE Healthcare, Chicago, IL, USA). Following electrophoresis, proteins were transferred in a semidry system onto a polyvinylidene difluoride (PVDF) membrane for 45 min at 0.1 mA. Membranes were blocked with 5% skim milk in Tris-buffered saline-Tween 20 (TBST) for 1h at room temperature. Membranes were then incubated with antibodies recognizing the gp120 (447-52D provided by the NIH AIDS Research and Reference Reagent Program) and Gag (p24 and p55) (p24 Antibody [24-4]; Santa Cruz Biotechnology, Santa Cruz, CA, USA). A goat anti-human horseradish peroxidase (HRP) (catalog no. sc-2907; Santa Cruz Biotechnology, Santa Cruz, CA, USA) and a chicken anti-mouse-HRP (catalog no. sc-2954; Santa Cruz Biotechnology, CA, USA) was used to detect gp120 and p55. Finally, Env and Gag proteins were detected with Clarity Western ECL (Bio-Rad, Hercules, CA, USA) using the ImageQuant LAS 500 imager (GE Healthcare, Chicago, IL, USA). Quantitative Western blot analysis was performed as described previously with a standard curve of gp120 recombinant protein (HIV-1 Bal) to quantify gp120 in VLP samples (28). The presence of trimers was analyzed by Western blotting in BN-PAGE gels as described previously (62). Briefly, VLPs were solubilized at room temperature for 10 min in 0.12% Triton X-100 in 1 mM EDTA-1.5 aminocaprotic acid with 1 μl of protease inhibitor cocktail (Sigma-Aldrich, St. Louis, MO, USA) at a 1:1 ratio. The sample buffer used was 100 mM morpholinepropanesulfonic acid (MOPS), 100 mM Tris-HCl, pH 7.7, 40% glycerol, and 0.1% Coomassie blue G250 added at a 1:1 ratio. Samples were loaded onto 4 to 12% Bis-Tris NuPAGE (Invitrogen, Carlsbad, CA, USA) separated for 3 h at 100 V at 4°C using high-molecular-weight calibration kit for native electrophoresis (Amersham, Buckinghamshire, UK) as a size standard. An AC10.29 rgp120 (gp120 monomer), a 96ZM651 SOSIP (trimer), and a JRFL-VLP, kindly provided by James Binley (VLP-incorporated trimer), were used as controls. Proteins were blotted in a wet tank onto a PVDF membrane at 4°C and 30V overnight. Then, membranes were blocked with 4% nonfat milk/PBS buffer for 1 h at room temperature and stained with an anti-gp120 and anti-gp41 antibody cocktail (MAbs 2G12, b12, 39F, 2F5, 4E10, and 447-52D, each at 1 μg/ml) overnight at 4°C. Finally, membranes were washed 3 times with PBS and probed by an anti-human-HRP (Jackson ImmunoResearch, West Grove, CA, USA), and proteins were detected by Pierce ECL Western blotting substrate (Thermo Fisher Scientific, Waltham, MA, USA).

### Rabbit immunization.

Five groups of rabbits were immunized (4 per group), HIV-1-Gag-VLPs as a control group (G_1_), HIV-1-Gag VLPs with 887 Env (G_2_), 887 SOSIPv8.2 (G_3_), HIV-1-Gag VLPs with 936 Env (G_4_), and 936 SOSIPv8.2 (G_5_). In all groups, Adjuplex adjuvant was used (Sigma-Aldrich, St. Louis, MO, USA). The v8.2 SOSIP version was used in this study, which incorporates 16 additional mutations from the original sequence. Two mutations expose the epitope of PGT145 (R156N and H158S). Seven others are so-called trimer-derived mutations (TDs). These mutations decreased aggregation and dissociation to dimers/monomers, in addition to achieving great thermostability. These changes were grouped into two important regions of the protein envelope, including the interface and the V2/V3 loops’ neither of them modified the antigenicity of these Envs (63). Finally, SOSIPsv.8.2 also contained 7 other mutations that increase affinity by bNAb PGT121 reverted to the germ line that have demonstrated their ability to activate PGT121 germ line B cells *in vivo* in mice (64).

The vaccination protocol used was a DNA prime (G_1_, pcDNA-dGag-250μg and pcDNA3.1-250μg, G_2_: pcDNA-dGag-250μg and pcDNA3.1-887-250μg; G_3_, pcDNA3.1-887-500μg; G_4_, pcDNA-dGag-250μg and pcDNA3.1-936-250μg; and G_5_, pcDNA3.1-936-500μg; 250 μg each; 500 μg total DNA/animal) followed by one DNA boost (500 μg/animal) and 3 VLP or SOSIP boosts (0.6 μg of gp120/animal/immunization for VLPs and 20 μg of SOSIP/animal/immunization). Animals were immunized at 3-week intervals. Seventeen weeks after immunization (2 weeks after the last boost), animals were exsanguinated. All rabbits were maintained in 785- by 840- by 1,840-mm cages with access to water and food *ad libitum* and under constant humidity and temperature with a light/dark cycle of 12 h. All procedures were conducted in accordance with the guidelines established by the University of Barcelona’s bioethics committee, as stated in Law 214/97 (July 30) drawn up by the Generalitat de Catalunya.

### gp120, VLP and SOSIP ELISAs.

Monomeric recombinant gp120 (HIV-1 Bal) was coated at 2 μg/ml in PBS in (ELISA)= wells (Inmulon; Thermo Fisher Scientific, Waltham, MA, USA) and incubated at 4°C overnight. Next, wells were blocked with 150 μl of 5% nonfat powdered milk in PBS at 37°C for 1 h. We added 100 μl of the corresponding sera dilutions in 5% PBS-milk to each well, and they were incubated at 37°C for 1 h. After washing three times with PBS plus 0.05% Tween 20, 100 μl of horseradish peroxidase-conjugated goat anti-rabbit immunoglobulin G antibody (catalog no. sc-2004; Santa Cruz Biotechnology, Santa Cruz, CA, USA) diluted 1:2,000 in 5% milk in PBS was added to each well, and the plates were incubated at 37°C for 1 h. Plates were then washed 6 times with PBS-Tween, and then 100 μl of tetramethylbenzidine reagent (Calbiochem, San Diego, CA, USA) was added to each well. Ten minutes later, 50 μl of hydrochloric acid was added to each well, and optical density at 450 nm was measured using a spectrophotometer (Sunrise; Tecan, Zürich, Switzerland). Gp120 reactivity for each group during the immunization process was compared to the group immunized with VLPs with no envelope with a two-way analysis of variance (ANOVA) Bonferroni posttest. ELISA endpoint titers at exsanguination were compared with a Mann-Whitney U test. For VLP ELISA, 50 μl of VLPs per well at 0.1 μg of gp120/ml were used to coat ELISA plates. Sera and MAb binding was then assessed by conducting an ELISA as described above, omitting detergent from PBS wash buffers, and probing with an anti-human or anti-rabbit immunoglobulin G antibody, as described above (65). For Ni-nitrilotriacetic acid (Ni-NTA) His-capture ELISA, the purified SOSIP trimers (1.0 μg/ml) were diluted in TBS (50 mM Tris-Cl, pH 7.6, 150mM NaCl) and immobilized on 96-well Ni-NTA ELISA plates for 2 h at room temperature (Qiagen). Subsequent steps were performed as previously described (11).

### IFN-γ quantification.

IFN-γ rabbit sera levels were quantified with RayBio rabbit IFN gamma kit (Norcross, GA, USA) following the manufacturer’s recommendations.

### Neutralization assays.

Serum samples from HIV infected individuals were initially tested using TZM-bl target cells as described previously, in triplicate at a 1;200 dilution with the six recombinant replication-competent viruses representing 5 different genetic subtypes, VI 191 (subtype A, tier 2), 92BR025 (subtype C, tier 1B), 92UG024 (subtype D, tier 2), CM244 (subtype AE, tier 2), NL4-3 (subtype B, tier 1A), and AC10.029 (subtype B, tier 2) (59). For rabbit sera neutralization, 1 ml of serum from immunized animals were preadsorbed with 4 × 10^7^ 293F cells (the same cells used for VLP production) for 1 h at room temperature. Next, IgGs from preadsorbed immunized animal sera were purified with protein A columns (GE Healthcare, Chicago, IL) and tested with a previously described virus minipanel as indicated above (25). Purified IgGs were also tested against pseudotyped viruses with 887-SOS-T and 936-SOS-T envelopes in a TZM-bl assay.
